# Study on the Technology of Laparoscopic Ovum Pick-Up and In Vitro Embryo Production in Chongming Goats

**DOI:** 10.3390/biology13090699

**Published:** 2024-09-06

**Authors:** Xiangli Wu, Dongxu Li, Ying Chen, Yangsheng Wu, Gulimire Abudureyimu, Wei Zhang, Kelu Deng, Zhen Huang, Jiapeng Lin, Liqin Wang

**Affiliations:** 1 Institute of Biotechnology, Xinjiang Academy of Animal Sciences, Urumqi 830000, China; wuxiangli2022@163.com (X.W.); cy08272024@163.com (Y.C.); xj_wys@126.com (Y.W.); gulimire127@163.com (G.A.); zw02112024@163.com (W.Z.); 2 College of Animal Science and Technology, Xinjiang Agricultural University, Urumqi 830000, China; 3 College of Animal Science and Technology, Nanjing Agricultural University, Nanjing 210095, China; 2022205013@stu.njau.edu.cn; 4 Liuji Yuanzhi Biotechnology Co., Ltd., Shanghai 202150, China; shljyzsw@163.com; 5 Wanhe Agricultural Technology Development Co., Ltd., Shanghai 202150, China; tomhuang@vertorganic.com

**Keywords:** laparoscopic ovum pick-up, cumulus–oocyte complexes, in vitro embryo production, ovarian follicular development

## Abstract

**Simple Summary:**

This study investigates the use of Chongming goats for optimizing laparoscopic ovum pick-up (LOPU) and in vitro embryo production (IVEP). Key metrics such as the recovery rate of cumulus–oocyte complexes (COCs), the number of ovarian follicles, and the quality of collected COCs are assessed to refine estrus synchronization and superovulation techniques. Additionally, the study examines the effectiveness of LOPU using indicators like cleavage and blastocyst rates to enhance IVEP efficiency, aiming to develop a robust LOPU technology system for small ruminants.

**Abstract:**

The application of laparoscopic ovum pick-up (LOPU) and in vitro production of embryos (IVEP) technologies has opened up a new path for purebred breeding and breed improvement in goats. However, due to the complexity of the procedures and multiple influencing factors, these technologies have not been widely adopted in goat production. This study explores factors affecting the efficiency of LOPU in goats by comparing the use of controlled internal drug release (CIDR) for estrus synchronization, conventional FSH versus long-acting recombinant ovine FSH (R-FSH) for superovulation, and the timing of LOPU at 48 h, 60 h, and 72 h of follicular development. The metrics evaluated included the recovery rate of cumulus–oocyte complexes (COCs), the average number of ovarian follicles, the average number of COCs, and the average number of available COCs. The results demonstrated that the efficiency of LOPU was significantly higher with two doses of R-FSH compared to the conventional FSH superovulation protocol and the control group (*p* < 0.05), with two doses of R-FSH providing a higher LOPU efficiency than one dose (*p* < 0.05). Using CIDR for estrus synchronization showed no significant difference in LOPU efficiency compared to the non-CIDR group. Similarly, the efficiency of LOPU showed no significant difference between the hormone treatments at 48 h, 60 h, and 72 h. By exploring and optimizing the factors influencing LOPU, we ultimately established a LOPU technology system for goats that meets the production needs of small ruminants.

## 1. Introduction

The rapid growth of intensive goat farming has increased the demand for high-quality meat goats, making efficient breeding techniques essential. Currently, techniques such as estrus synchronization, artificial insemination, and multiple ovulation and embryo transfer (MOET) are widely used in goat breeding [1,2]. Significant progress has also been made in technological systems such as ovum pick-up (OPU), in vitro embryo production (IVEP), superovulation in young animals, gene editing, and cloning [3,4,5]. Following the development model of the ruminant cattle industry, the number of IVEP and embryos transferred has exceeded the number of IVEP, and the number is still increasing, which is a trend that shows that the livestock breeding industry has begun to shift from traditional MOET to IVEP. This shift serves as an important reference for the future development of IVEP in the goat industry.

IVEP mainly includes in vitro oocyte maturation (IVM), in vitro fertilization (IVF), and in vitro culture of embryos (IVC) [6,7]. Oocytes for IVEP are isolated from fresh ovaries in abattoirs or harvested in vivo. Compared to in vitro ovarian isolation, OPU has several advantages [8]. These advantages include overcoming the uncertainties associated with obtaining oocytes from abattoirs (such as genealogy, age, and health), shortening the generation interval, accelerating the expansion of superior breeding stock, and utilizing oocytes from females with reproductive disorders. The methods of OPU currently used in production include traditional surgical laparotomy oocyte collection, ultrasound-guided oocyte collection, and laparoscopic ovum pick-up (LOPU).

The traditional surgical laparotomy oocyte collection method involves surgically exposing the ovaries of the donor ewes to the external environment and then using a harvesting needle to visually aspirate the ovarian follicles to obtain the oocytes. This procedure causes significant damage to the donor’s reproductive system and greatly shortens the lifespan of the ewes [9,10]. The ultrasound-guided oocyte collection method uses B-mode ultrasound to assess the quality of the ovarian follicles in the donor female employing different reflected wave intensities. The follicle images are used to collect the oocytes by puncture and recover them using negative pressure, a method that is widely used in the production of cattle, horses, and other medium and large animals, but it is not suitable for the production of goats [11,12]. The LOPU method combines puncture needles and endoscopes to collect oocytes from donor females. The location of the ovarian follicles and their developmental status can be observed through the endoscope. This surgical method greatly reduces harm to the female animal and has become the preferred method for obtaining oocytes from small ruminants [13].

It has been demonstrated that LOPU is less invasive, does not cause postoperative complications, can be performed several times in a short period, and does not affect the developmental capacity of the COCs [14,15,16]. Furthermore, in addition to their application to IVEP, LOPU-derived oocytes have been successfully applied to somatic cell nuclear transfer [17] and microinjection for the production of transgenic animals [18,19]. LOPU is widely used on ruminants such as goats and sheep [20], deer [21], young buffaloes [22], and dairy cows [23]. Therefore, LOPU has become an effective alternative to traditional MOET. However, uncertainty in the quantity and quality of oocytes still limits the large-scale use of the technique. The variability depends on factors related to the donor, such as the breed, age, and individual response of the donor [24], and other controllable aspects, such as hormonal stimulation, length or type of needles, aspiration pressure, and proficiency of the operator. Therefore, the large-scale application of LOPU technology still faces significant challenges.

In this study, we investigated the effects of hormone use, the placement of the CIDR, and the timing of follicle development on the efficiency of LOPU of Chongming white goats. Moreover, IVEP and embryo transfer were carried out to verify the feasibility of LOPU–IVEP for goat breeding. The aim was to improve the efficiency of LOPU–IVEP in goats and establish a LOPU technology system for goats that meets the production needs of small ruminants.

## 2. Material and Methods

### 2.1. Ethics Statement

**Figure 1:**
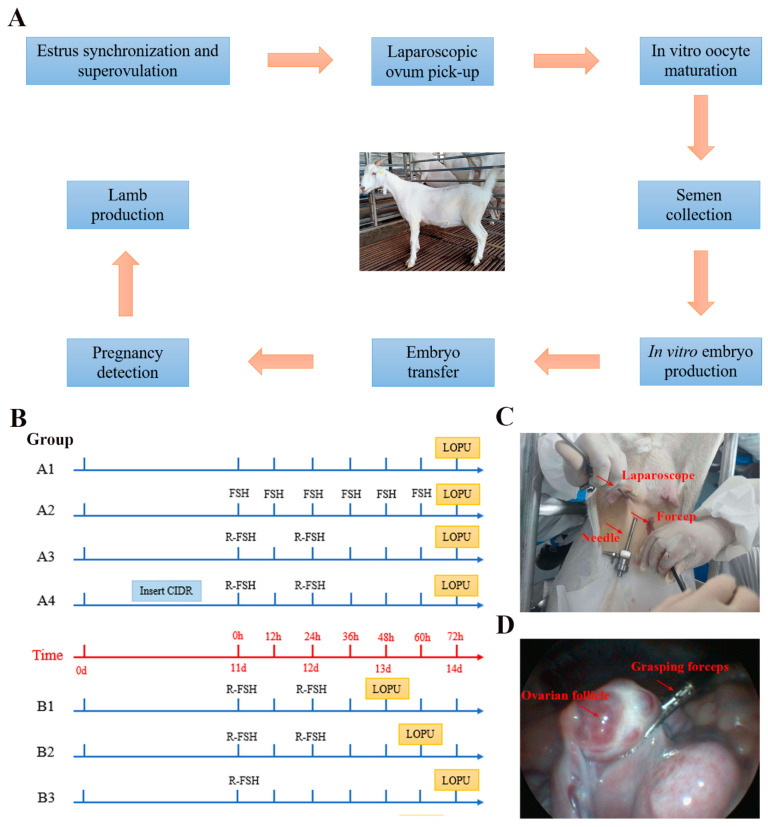
The experimental procedures of laparoscopic ovum pick-up (LOPU) and in vitro embryo production (IVEP) technologies. (**A**) Schematic diagram of LOPU and IVEP steps. (**B**) Goat superovulation protocols in each group. (**C**) LOPU of goats with abdominal puncture. (**D**) Ovarian fixation in LOPU.

The experimental procedure of LOPU and IVEP technologies is shown in Figure 1A. Two-year-old healthy multiparous goats were selected from Wanhe White Goat Breeding Farm in Jiangsu. The reproductive tracts of all goats were confirmed to be healthy using ultrasonography, with no signs of purulent or mucoid vaginal secretions, clinical or subclinical endometritis, reproductive system diseases, microbial contamination, or ovarian cysts. All protocols involving the use of animals adhered to the approved Guidelines for Animal Experiments of Nanjing Agricultural University and received approval from the Animal Care and Use Committee of Nanjing Agricultural University (Approval ID: SYXK2022-0031).

### 2.2. Experimental Design

#### 2.2.1. Effect of Different Superovulation Regimens on LOPU Efficiency

In experiment 1, three superovulation regimens were compared. Group A1: blank control (*n* = 13); Group A2: intramuscular injection of 300 IU FSH (Sansheng, Ningbo, China) twice daily in a decreasing dose over 3 days (60/60, 50/50 and 40/40 IU) (*n* = 4); Group A3: intramuscular injection of long-acting recombinant ovine FSH (R-FSH) (Youliankang Pharmaceutical Technology Co., Ltd., Guangzhou, China) 8 mg twice in total with an interval of 24 h (*n* = 13). LOPU was performed at 72 h after treatment, and the COC recovery rate, the average number of ovarian follicles, the average number of COCs, and the average number of available COCs were counted (Figure 1B).

#### 2.2.2. Effect of Intravaginal Controlled Internal Drug Release on LOPU Efficiency

In experiment 2, two comparison groups were set up. Group A3: control group (*n* = 7); Group A4: treatment group with the addition of intravaginal CIDR, lasting for 11 days, with the same superovulation regimens as Group A3 (*n* = 6). Intramuscular injection of R-FSH 8 mg twice in total with a 24 h interval. LOPU was performed 72 h after treatment, and the COC recovery rate, the average number of ovarian follicles, the average number of COCs, and the average number of available COCs were counted (Figure 1B).

#### 2.2.3. Effect of Follicular Development Period on the Efficiency of LOPU

In experiment 3, three comparative groups were set up without placing intravaginal CIDR. Group B1: LOPU was performed after 48 h of stimulation with 8 mg twice in total of R-FSH intramuscular injection (*n* = 4). Group B2: the procedure was performed after 60 h of stimulation with R-FSH (*n* = 6). Group A3: the procedure was performed after 72 h of stimulation with R-FSH (*n* = 13). The COC recovery rate, the average number of ovarian follicles, the average number of COCs, and the average number of available COCs were counted (Figure 1B).

#### 2.2.4. Effect of Hormone Treatment Mode on LOPU Efficiency

In Experiment 4, two comparison groups were set up under the condition that the total amount of hormones used in superovulation was the same. Group A3: intramuscular injection of R-FSH 8 mg twice in total with an interval of 24 h (*n* = 13). Group B3: 16 mg of R-FSH injected at one time on day 11 (*n* = 4). LOPU was carried out 72 h after superovulation, and the COC recovery rate, the average number of ovarian follicles, the average number of COCs, and the average number of available COCs were counted (Figure 1B).

### 2.3. Laparoscopic Ovum Pick-Up

Preoperative treatment: the donor goat was fasted for 24 h before surgery and anesthetized with 0.12 mL/kg anesthetic mixture through intramuscular injection (Su-Mian-Xin II or Xylazine Hydrochloride Injection, the ingredient is 100 mg/mL xylazine; Institute of Military Veterinary, Changchun City, China). The goat was fixed on a surgical frame with its head tilted downwards at 45° and covered with a bandage after disinfection.

LOPU Procedure: A puncture was made at the base of the breast at the midpoint of the right and left inguinal junctions for the placement of the ovarian needle trocars and non-invasive grasping forceps (Huida Medical Equipment Co., Ltd., Hangzhou, China). In addition, a puncture was made at the base of the breast at a distance of about 15 cm cephalad for the placement of the laparoscope (Bewick Biotechnology LLC, Beijing, China) so that the three-point line became an equilateral triangle (Figure 1C). The bladder was positioned under laparoscopic observation, and then the uterus was searched downwards to locate the ovaries. After the grasping forceps secured the ovaries, they were gently turned over to ensure that all visible ovarian follicles were exposed to view (Figure 1D), and oocytes were collected by aspiration with the ovum-pumping needle into a BD 14 mL tube (BD Falcon, Corning, NY, USA). Immediately after oocyte collection, the tubes were sent to the laboratory where oocytes were picked up under a stereomicroscope (SMZ-645; Nikon, Tokyo, Japan) and graded for quality. Oocyte collection sequence: starting from the fixed site of the fixed ovary, the oocytes were collected gradually upwards, from small to large ovarian follicles. It took roughly 10 to 15 min to harvest both ovaries, at the end of which the two non-invasive ovarian fixation forceps and two trocars were removed before the laparoscope was removed.

Postoperative management: a dilute solution of penicillin (Ampicillin Sodium for Injection, Harbin Pharmaceutical Group Co., Ltd. General Pharm. Factory, Harbin, China) was injected through the laparoscopic perforator to prevent ovarian adhesions from becoming infected, after which the perforator was withdrawn from the wound site, sterilized, and de-anesthetized.

### 2.4. Grading of Oocyte Quality

**Figure 2:**
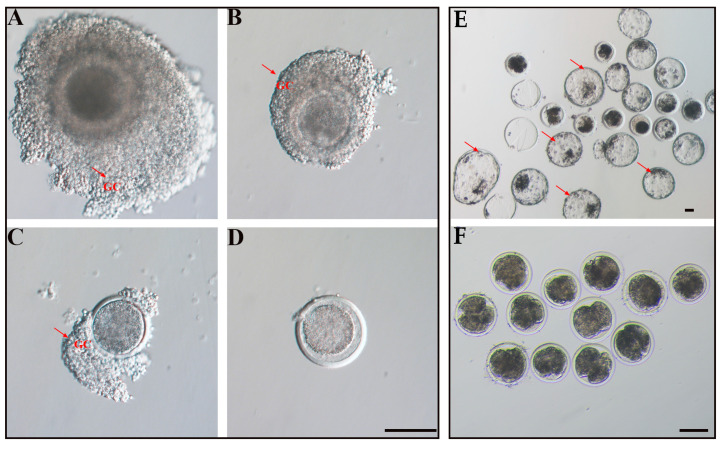
Oocyte quality classification and early embryonic development. (**A**) Homogeneous cytoplasm, encapsulated by at least 3 layers of granulosa cells is defined as grade A; (**B**) encapsulated by 1–2 layers of granulosa cells is defined as grade B; (**C**) homogeneous cytoplasm but not evenly encapsulated by granulosa cells is defined as grade C; (**D**) inhomogeneous cytoplasm or semi-transparent cytoplasm, dead or degenerate oocytes is defined as grade D. Scale bar, 100 µm. Arrows indicate ovarian granulosa cells. Development of embryos in vitro at 48 h (**E**) and 168 h (**F**). Scale bar, 100 µm. Arrows indicate blastocysts.

Oocytes were classified into 4 grades according to the morphological characteristics of the COCs. Grade A: homogeneous cytoplasm, encapsulated by at least 3 layers of granulosa cells (Figure 2A); Grade B: homogeneous cytoplasm, encapsulated by 1–2 layers of granulosa cells (Figure 2B); Grade C: homogeneous cytoplasm but no granulosa cells, naked oocytes (Figure 2C); Grade D: inhomogeneous cytoplasm or semi-transparent cytoplasm, dead or degenerate oocytes (Figure 2D). Grade A oocytes were defined as usable oocytes for subsequent culture.

### 2.5. In Vitro Embryo Production (IVEP)

The IVEP procedure in goats was described in previous studies [25]. In vitro oocyte maturation (IVM): oocytes of grades A and B isolated as described above were washed more than three times with IVM medium, transferred into four-well dishes (Thermo Scientific, Roskilde, Denmark) containing 600 µL of IVM (BO-IVM, IVF Bioscience, Falmouth, UK) and 300 µL of oil (Sigma-Aldrich, St. Louis, MO, USA), and cultured in an incubator at 38.5 °C, 5% CO_2_, and 100% humidity for 22–24 h. After maturation, oocytes were used for subsequent fertilization.

In vitro fertilization (IVF): frozen sperm was thawed quickly in sterile water at 38 °C and subsequently tested for viability. When sperm viability exceeded 40%, the sperm was added to pre-heated PureSperm liquid (IVF Bioscience) and centrifuged at 300× *g* for 5 min. At the end of centrifugation, the supernatant was aspirated with a micropipette and resuspended to 2 to 9 × 10^6^ spermatozoa/mL in an IVF (IVF Bioscience) culture medium. Sperm was added to the fertilized micro-droplet, and a gentle rotation of the oocyte, driven by sperm, could be observed under the microscope. The oocytes were incubated at 38.5 °C, 5% CO_2_, and 100% humidity for 12–16 h.

IVEP: after fertilization, granulosa cells were removed by hyaluronidase (Sigma-Aldrich, St. Louis, MO, USA), transferred into pre-heated IVC (IVF Bioscience) culture drops, and cultured in an incubator with 38.5 °C 5% CO_2_, 5% O_2_, 90% N_2_, and 100% humidity. The cleavage rate was examined at 48 h (Figure 2E), and the blastocyst rate was recorded 168 h after IVC (Figure 2F).

### 2.6. Embryo Transfer

The steps of embryo transfer were described in previous studies [26]. In short, recipients were treated with the same hormones as donors to ensure similar physiological states. Using laparoscopy to view the corpus luteum on both ovaries, the blastocysts were transferred to the uterine horn on the side with the corpus luteum. Pregnancy detection was performed 35–40 days after transfer. Conventional recipients are defined as ewes that underwent identical hormonal treatments, timed to coincide precisely with those administered to the donor ewes. Special recipients refers to donor ewes that underwent LOPU and were subsequently utilized as recipients for the transplantation process.

### 2.7. Statistical Analysis

It was determined that the distribution of all data accorded with normal distribution by the Kolmogorov–Smirnov goodness-of-fit test. If data were not normally distributed, they were log-transformed and retested for normality before analysis. Subsequently, statistical analyses were subjected to one-way analyses of variance (ANOVA) run using Duncan’s test or Tukey test. Analysis was conducted using SPSS v27.0 (Chicago, IL, USA). These data were presented as the mean ± SE and considered statistically significant when *p* < 0.05.

## 3. Results

### 3.1. Effect of Different Superovulation Regimens on LOPU Efficiency

As shown in Table 1, there were no significant differences in COC recovery rates among the blank control group A1, the conventional FSH group A2, and the R-FSH group A3. However, the average number of ovarian follicles, the average number of COCs, and the average number of viable COCs in the R-FSH group A3 were significantly higher than those in the conventional FSH group A2 and the blank control group A1, and group A2 was significantly higher than group A1.

### 3.2. Effect of Intravaginal Controlled Internal Drug Release on LOPU Efficiency

Given the experimental results of experiment 1, we chose the group A3 superovulation protocol to investigate the effect of the CIDR on the efficiency of LOPU. By comparing the CIDR-placed A3 group with the unplaced group A4, there were no significant differences in the average number of ovarian follicles, average number of COCs, or average number of available COCs (Table 2).

### 3.3. Effect of Follicular Development Period on the Efficiency of LOPU

The superovulation protocol without the placement of the CIDR was performed according to reference A3, with LOPU at 48 h (Group B1), 60 h (Group B2), and 72 h (Group A3) after the first injection of R-FSH. The results demonstrated no significant differences in average COC recovery rates, number of ovarian follicles, average number of COCs, or average number of available COCs between groups B1, B2, and A3 (Table 3).

### 3.4. Effect of Hormone Treatment Mode on LOPU Efficiency

The effect of the hormone injection mode on LOPU was investigated by ensuring that no CIDR was placed and that the total amount of hormone injected and the time of the LOPU were the same. The results demonstrated no significant differences in the average COC recovery rates between the two-injection mode group A3 and the single-injection mode group B3 (Table 4). However, the average number of ovarian follicles, the average number of COCs, and the average number of viable COCs in the two-injection mode group A3 were significantly higher than those in the conventional FSH group A2 and the single-injection mode group B3.

### 3.5. IVEP and Embryo Transfer in Goats

Based on the efficiency of LOPU in the experiments mentioned above, we chose to place the CIDR for estrus synchronization, and superovulation with two injections of R-FSH totaling 16 mg, and we performed LOPU after 48 h of ovary development. Sixteen Chongming goats were selected as donors, and a total of 359 available COCs were collected by LOPU (Table 5). After IVF, a total of 304 oocytes were cleaved, and 193 developed into blastocysts. Subsequently, 102 blastocysts were transferred into the uterine horns of 34 recipients, with three blastocysts per goat. Twenty-two transplants were performed on conventionally treated recipients, resulting in a pregnancy rate of 63.63%, while 12 transplants were performed on specially treated recipients (goats with LOPU), resulting in a pregnancy rate of 58.33% (Table 6).

## 4. Discussion

The ability to efficiently obtain large quantities of high-quality oocytes without serious damage to maternal reproduction through LOPU has become a crucial method for obtaining oocytes from small ruminants [27]. However, LOPU has not been widely promoted in goat IVEP due to several important reasons. These reasons include the difficulty of the technical operation, which requires a combination of endoscopes and oocyte extraction needles, the type of oocyte collection needle used [28], and the pressure of the aspiration pump [29]; all have to be adjusted according to individual differences. Additionally, factors such as estrus synchronization, superovulation treatment [30,31], and physiological differences among ewes play a significant role [32]. Therefore, this study explores an efficient and stable LOPU technology system by optimizing variable factors such as synchronization of estrus, superovulation treatment, and follicle development time in goats.

Currently, many hormone stimulation programs are used in goat production. These programs begin with the synchronization of estrus and gonadotropin stimulation, which allows the simultaneous recruitment of a large number of ovarian follicles. The synchronization of estrus programs generally involves the use of progesterone-containing release devices that are placed in the vagina and usually remain there for 10–14 days until the ovarian follicles are aspirated. Although progesterone administration during follicular growth does not affect the number of ovarian follicles, it does increase cleavage rates and embryonic development. Studies have shown that high progesterone concentrations during ovarian hormone stimulation in cattle result in higher-quality embryos collected seven days after estrus [33,34]. The use of a vaginal pessary containing progesterone, both with and without progesterone, in sheep before OPU showed that COC recoveries per sheep were not affected by progesterone treatment, but progesterone-treated ewes had significantly higher cleavage and blastocyst rates [35]. In our study, similar results were found; there were no significant differences in COCs recovery (%), average number of ovarian follicles, average number of COCs, and average number of viable COCs in LOPUs of ewes with the CIDR compared to those without the CIDR.

FSH is the hormone of choice for ovarian stimulation in superovulation and embryo transfer in goats. Given the stringent requirements for follicular development in LOPU, the appropriate hormone dose is significant. If the dose of FSH hormone is too low, the ovaries may respond only slightly; if the dose is too high, the blood flow through the ovaries increases, making OPU more difficult. It has been shown that a high dose of FSH leads to a significant increase in the number of large ovarian follicles and an increase in intra-ovarian pressure, which may lead to leakage of follicular fluid and oocytes during follicular puncture [36]. Additionally, higher concentrations of FSH can reduce the ovarian response to FSH by lowering the mRNA expression of FSH receptor levels in the ovarian follicles, thus affecting the efficiency of oocyte collection [37].

Six injections of pFSH, totaling 180 mg, were given at 12 h intervals before the withdrawal of the CIDR, resulting in an oocyte recovery rate of 81.2% in goats with an average of 27.0 ± 5.0 visible ovarian follicles per goat, 21.9 ± 4.1 ovarian follicles per goat, a fertilization rate of 85.6%, and a cleavage rate of 82.4% [24]. Similarly, decreasing injections of ewes using 180 mg of Canadian-made pFSH yielded an average of 13.3 ± 1.8 oocytes per ewe [38]. Six injections of the same dose of FSH totaling 175 IU at 12 h intervals before withdrawal resulted in an average of 26.69 ± 3.66 oocytes per sheep [39]. In this study, we set up a blank control group, a group receiving the conventional FSH regimen of decreasing doses, and a group receiving two long-acting recombinant FSH doses. The results demonstrated that the two long-acting recombinant FSH doses had the highest efficiency in LOPU, while the blank control group had the lowest. At the same time, we also compared the effect of different injection methods on the efficiency of LOPU under the circumstance of ensuring the total amount of hormones. The results showed that with two injections, the efficiency of LOPU was significantly better than with one injection.

Mammalian oocytes develop and mature within the ovarian follicle, and their developmental potential is closely linked to the growth and fate of these follicles; oocytes from larger follicles have a greater developmental potential to reach the blastocyst stage in vitro [40,41]. It has been shown that goat oocytes collected 36 h after PMSG treatment are still in the immature stage and require 27 h of IVM culture to become meiotic [18]. LOPU can be performed at 10 h, 24 h, 36 h, or 48 h after gonadotropin injection [42]. Extending the time interval between the last FSH injection and LOPU has been shown to improve oocyte production and quality in goats, with LOPU at 60 h or 72 h after the last FSH injection yielded better quality oocytes compared to the 36 h interval [43]. In this study, the results demonstrated no significant differences in the average COC recovery rates, number of ovarian follicles, average number of COCs, or average number of available COCs between groups B1, B2, and A3.

Based on the efficiency of LOPU in the experiments mentioned above, we chose to place the CIDR for the estrus synchronization and superovulation with two injections of R-FSH totaling 16 mg. Then, LOPU was performed after 48 h of ovary development. After obtaining oocytes, those covered by more than three layers of granulosa cells were selected for IVF and IVC. The number of oocyte layers was extremely important for IVM and even for IVF and IVC of embryos [31,42]. To improve the success of the embryo transfer, an intrauterine environment appropriate to the stage of embryo development should be provided, which was achieved in this study by the use of hormones that induced a state of synchronization between the donor and the recipient. The embryos were transferred into the uterine horns ipsilateral to the corpus luteum by the laparoscopic method. This method has been reported in previous studies [44]. However, the innovative aspect of this experimental embryo transfer is the use of two different types of recipients to explore the pregnancy rate of the transferred ewes.

## 5. Conclusions

This study found that placing the CIDR for estrus synchronization, administering two doses of long-acting recombinant ovine FSH totaling 16 mg for superovulation, and performing LOPU at 48 h of follicular development was an effective and feasible method for LOPU-IVEP in goats. This study aimed to improve the efficiency of LOPU-IVEP in goats and establish a technical system for LOPU in small ruminants to meet production needs.

## Figures and Tables

**Table 1:** Effect of hormones on LOPU efficiency.

Group	No. of Ovarian Follicles	No. of COCs	No. of Available COCs	COs Recovery Rate (%)	Average No. of Ovarian Follicles	Average No. of COCs	Average No. of Available COCs
A1	113	104	76	96.00 ± 5.65	8.69 ± 0.77 ^a^	8.00 ± 2.16 ^a^	5.85 ± 0.41 ^a^
A2	71	73	69	102.50 ± 5.31	17.75 ± 0.62 ^c^	18.25 ± 2.99 ^c^	17.25 ± 1.03 ^c^
A3	297	333	311	112.77 ± 6.52	22.85 ± 0.66 ^b^	25.62 ± 4.99 ^b^	23.92 ± 1.50 ^b^

Note: A1: blank control group (*n* = 13); A2: conventional FSH group (*n* = 4); A3: long-acting recombinant ovine FSH group (*n* = 13). ^a,b,c^ Values with different superscript letters within individual columns are statistically different (*p* < 0.05).

**Table 2:** Effect of intravaginal controlled internal drug release on LOPU efficiency.

Group	No. of Ovarian Follicles	No. of COCs	No.of Available COCs	COC Recovery Rate (%)	Average No. of Ovarian Follicles	Average No. of COCs	Average No. of Availabile COCs
A3	146	160	131	107.81 ± 10.57	20.86 ± 1.74	22.86 ± 3.18	20.00 ± 3.05
A4	136	138	129	103.57 ± 7.20	22.67 ± 1.69	23.00 ± 1.34	22.17 ± 2.30

Note: A3: control group (*n* = 7); A4: treatment group with the addition of intravaginal CIDR (*n* = 6).

**Table 3:** Effect of follicular development period on the efficiency of LOPU.

Group	No. of Ovarian Follicles	No. of COCs	No. of Available COCs	COC Recovery Rate (%)	Average No. of Ovarian Follicles	Average No. of COCs	Average No. of Available COCs
A3	297	287	267	96.87 ± 1.96	22.85 ± 0.52	22.08 ± 0.50	20.54 ± 0.39
B1	94	91	85	96.95 ± 4.01	23.50 ± 065	22.75 ± 0.85	21.25 ± 0.94
B2	142	133	126	93.69 ± 1.73	23.67 ± 0.84	22.17 ± 0.87	21.00 ± 0.52

Note: Group B1: LOPU was performed after 48 h of R-FSH intramuscular injection (*n* = 4); Group B2: LOPU was performed after 60 h (*n* = 6); Group A3: LOPU was performed after 72 h (*n* = 13).

**Table 4:** Effect of hormone treatment mode on LOPU efficiency.

Group	No. of Ovarian Follicles	No. of COCs	No. of Available COCs	COC Recovery Rate (%)	Average No. of Ovarian Follicles	Average No. of COCs	Average No. of Available COCs
A3	297	287	267	96.87 ± 1.97	22.85 ± 0.52 ^a^	22.08 ± 0.50 ^a^	20.54 ± 0.39 ^a^
B3	80	73	70	91.25 ± 5.15	20.00 ± 0.41 ^b^	18.25 ± 1.11 ^b^	17.50 ± 1.19 ^b^

Note: Group A3: intramuscular injection of R-FSH 8 mg twice in total (*n* = 13); Group B3: 16 mg of R-FSH injected at one time (*n* = 4). ^a,b^ Values with different superscript letters within individual columns are statistically different (*p* < 0.05).

**Table 5:** In vitro embryo production of goats.

No. Recipients	No. Ovarian Follicles	Available COCs	No. Cleavage	Cleavage Rate (%)	No. Blastocyst	Blastocyst Rate (%)	Average No. of Blastocysts
16	361	359	304	84.68	193	63.48	12.06

Note: Blastocyst rate = number of blastocysts/number of cleavage.

**Table 6:** Pregnancy rates following embryo transfer.

Receptor Type	No. Recipients	No. Pregnancies	Pregnancy Rate (%)
Conventional recipients	22	14	63.63
Special recipients	12	7	58.33

## Data Availability

The data used to support the findings of this study are included in the article.
